# Relationship between workplace bullying, spirituality, and job burnout in paediatric nurses: A cross‐sectional study

**DOI:** 10.1002/nop2.1645

**Published:** 2023-02-15

**Authors:** Ying Fan, Mi Cao, Yumei Zhou, Peng Duan, Limin Xing

**Affiliations:** ^1^ Xiangyang No.1 People's Hospital Hubei University of Medicine Xiangyang City China; ^2^ Hubei Polytechnic Institute Xiaogan China

**Keywords:** job burnout, paediatric nurse, workplace bullying, workplace spirituality

## Abstract

**Aims:**

To investigate the relationships and pathways between workplace bullying, workplace spirituality, and job burnout in Chinese paediatric nurses.

**Design:**

A cross‐sectional descriptive survey was conducted with paediatric nurses from six tertiary hospitals in Hubei Province, China.

**Methods:**

The study consisted of 402 paediatric nurses. The data were collected using a sociodemographic data questionnaire, Negative Acts Questionnaire‐Revised, Maslach Burnout Inventory‐General Survey and Workplace Spirituality Scale. The model was tested using path analysis techniques within structural equation modelling.

**Results:**

Workplace bullying had positive and direct effects on the job burnout of paediatric nurses. Workplace spirituality partially mediated the relationship between workplace bullying and burnout.

**Patient or Public Contribution:**

Workplace spirituality may reduce the incidence of work bullying and job burnout in paediatric nurses. Nursing managers need to consider and cultivate the workplace spirituality of paediatric nurses, with the aim of creating a healthy working environment and ensuring the stability of the nursing team.

## 1 INTRODUCTION

Bullying in the workplace has been defined as “a situation where someone is subjected to social isolation or exclusion, his or her work and efforts are devalued, he or she is threatened, derogatory comments about him or her are said behind his or her back, or other negative behavior aimed to torment, wear down, or frustrate occur” (Kivimäki et al., 2000). Bullying in the workplace has become a serious social problem ever since workplace competition increased in the 21st century, and bullying in the nursing profession is becoming more frequent (Kim et al., 2019). As early as 100 years ago, bullying already existed in the nursing profession. In 1909, the “New York Times” published an article about nursing managers who abused their powers to openly persecute nurses, which aroused people's attention to workplace bullying in the nursing profession (“The New York Times”, 1909, p. SM8). Today, bullying is not a new issue. Although bullying has been a well‐researched topic in the past 30 years, it is unfortunately still widespread in the nursing profession.

## 2 BACKGROUND

Evidence showed 65% of nursing professionals in the USA observed lateral violence among co‐workers (Teo et al., 2021). A cross‐sectional survey of workplace bullying by clinical nurses showed that 39.59% of nurses were bullied in Beijing, China (Chen & Zheng, 2020). These statistics highlight the severity of workplace bullying in the nursing industry.

Paediatric nurses care for infants and young children who have not yet fully developed their language functions. Consequently, it is more difficult for paediatric nurses to communicate effectively with children and their caregivers. In addition, particular characteristics of the hospital environment may lead to adverse reactions in children, such as crying and fear (Ramsdell et al., 2016). At the same time, when a child is seriously ill, it may be a devastating blow to the entire family. The family of the child has experienced tremendous changes, such as destruction of family life and forced separation from important family members (Marques, 2017). Caregivers of children are prone to anxiety, depression, paranoia, emotional agitation, and other negative emotions (Katz et al., 2018) and may even act aggressively to nurses with complaints, insults, and beatings (Pan et al., 2018). Chen's survey showed that 215 of 304 paediatric nurses had experienced workplace violence, with an incidence rate of 70.7% (Chen et al., 2011). Paediatric nurses suffer more severe workplace bullying than nurses in other departments in China (Lin et al., 2017).

Studies have shown that continuous exposure to stressful environments caused by workplace bullying can increase the risk of hypertension and heart disease in nurses (Sauer & McCoy, 2017). Workplace bullying causes not only physical problems (such as physical discomfort, fatigue, and angina) (Laschinger et al., 2010), but also mental health problems (such as anxiety, depression, and post‐traumatic stress disorder) (Hamre et al., 2020). Workplace bullying also leads to job‐related problems, such as decreased job satisfaction in nurses, poor job performance, impaired working relationships, job burnout, and increased turnover tendency (Kim et al., 2019; Yun & Kang, 2018). For example, it causes nurses to lose professional confidence, affects the quality of nursing services, and increases the factors that negatively affect patient safety (Laschinger, 2014). In addition, it not only affects victims directly but also affects people who have witnessed bullying incidents, as they may also experience similar physical discomfort and psychological barriers (Kim et al., 2019). In short, workplace bullying increases the mobility of nurses and hinders the development of the organization.

In order to improve China's population structure and actively respond to an aging population, China promoted a new optimized ‘three‐child’ family planning policy on May 31, 2021, which allows a couple to have three children. Given the implementation of this policy, the demand for paediatric nurses will further increase. Therefore, improving the morale of paediatric nurses and reducing the turnover rate is of great significance to team stability.

The transactional theory of stress was proposed by Lazarus and Folkman in 1984 (Chen et al., 2019), and it is a pressure theory based on evaluation. They believe that pressure is the interaction between people and the environment. The source of pressure is based on the subjective perception of the individual. Pressure will only occur when the situation is beyond the resources of the individual. When assessing a situation involving human and environmental interactions, there are two important evaluation processes. (1) The primary evaluation is to assess the situation faced, to judge whether it represents a threat to the individual. (2) The secondary evaluation is based on the individual's own internal and external coping resources. The primary and secondary evaluations are combined to determine whether the environmental interaction is of great significance to the individual. After determining whether the pressure is obstructive or challenging, the individual responds through cognitive or behavioural changes (Folkman et al., 1986).

Based on the transactional theory of stress, we believe that paediatric nurses will regard workplace bullying as a source of pressure, and it is an important factor leading to job burnout in nurses (Oyeleye et al., 2013; Zhou et al., 2017). If the individual can correctly recognize or evaluate the pressure, the negative impacts of the pressure on the individual can be reduced. Workplace spirituality is about finding positive meaning in work and a feeling of connectedness expressed in interrelationships between colleagues and a healthy workplace atmosphere (Pirkola et al., 2016). It can have a significant impact on an individual's cognition and pressure evaluation, which is conducive to positively strengthening their impulse to counter pressure, stimulating their work efforts and increasing work enthusiasm. At the same time, inhibiting the negative impacts of obstructive pressure weakens the employees' sense of job burnout and reduces the turnover rate (Xuan et al., 2020). Employees with a high level of workplace spirituality can experience extraordinary meaning in their work. The sense of interconnection that the work brings to themselves, others, and the organization will affect the employees' cognitive behaviour (Zhang et al., 2017). Studies have shown that workplace spirituality is positively correlated with the employees' degree of job satisfaction and organizational harmony, and negatively correlated with work pressure and job burnout (Dal Corso et al., 2020; van der Walt & de Klerk, 2014).

**FIGURE 1:**
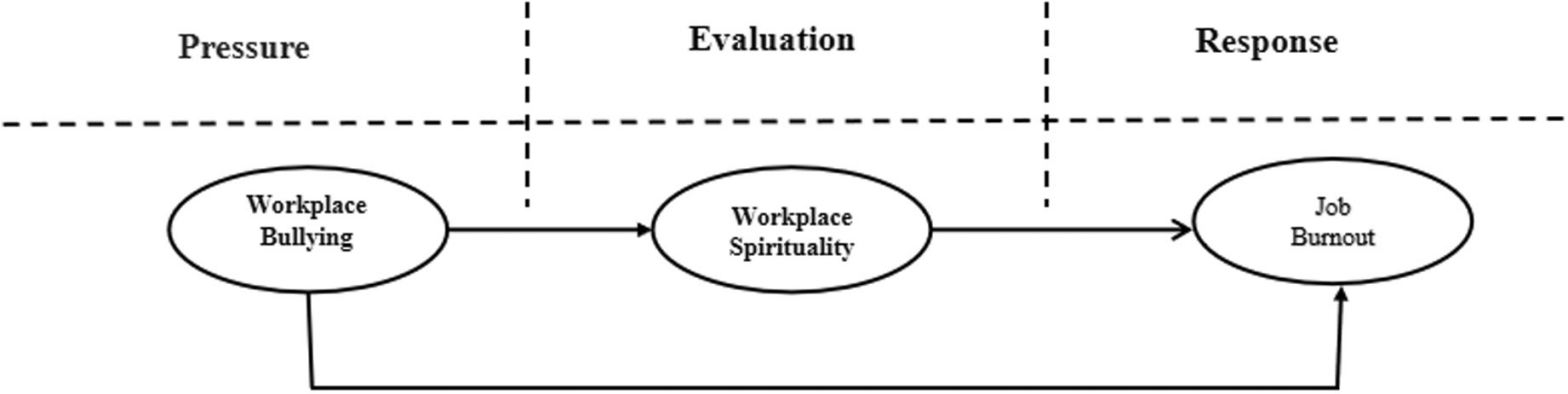
Hypothesized study model.

In view of these correlations, we constructed the theoretical model of this research study, as shown in Figure 1, based on the transactional theory of stress and previous related research studies. The hypotheses of our research are as follows:

Hypothesis 1 Workplace bullying is positively and directly related to burnout.

Hypothesis 2 Workplace bullying is negatively and directly related to workplace spirituality.

Hypothesis 3 Workplace spirituality is negatively and directly related to burnout.

Hypothesis 4 Workplace spirituality has a mediating effect between workplace bullying and burnout.

## 3 METHODS

### 3.1 Study design and participants

This study had a web‐based, cross‐sectional design with paediatric nurses as the target group and was conducted in six tertiary hospitals located in Hubei Province, China. Convenience sampling was employed to select the research samples in June 2021. The inclusion criteria were as follows: (1) nurses who had obtained professional registration, (2) nurses who had worked in paediatrics departments for more than 6 months, (3) no mental illness in the past or present, and (4) an ability and willingness to participate in this study. Paediatric nurses who could not participate in this study due to holidays, personal leave, or physical reasons were excluded. While there are differing views as to the optimal sample size for structural equation modelling (SEM), a generally accepted guideline from the literature is a sample size of 200 or more cases (Kline, 2005).Considering a non‐response rate of 20%, the final sample size required for this study was 240. Thus, the final 402 participants met this requirement.

### 3.2 Data collection

The researchers explained the purpose of the study to the nursing department staff at six hospitals, requested approval to perform the study, and collected data from the six approved hospitals. The directors of the nursing departments were requested to distribute the links of the internet survey to the paediatric nurses in each hospital, along with an explanatory statement, consent forms, and four questionnaires. The internet surveys can only be filled in once for each ID. For the anonymous surveys, we obtained informed consent from all the participants. The participants took approximately 20 min to complete the questionnaire.

### 3.3 Measures

#### 3.3.1 Demographics questionnaire

The demographic characteristics of our self‐designed questionnaire covered: gender, age, marital status, years of experience, employment type, education, title, and shift pattern.

#### 3.3.2 Workplace bullying

The Chinese version of the Negative Acts Questionnaire‐Revised (NAQ‐R), originally developed by Einarsen et al. (2009), was translated into Mandarin Chinese by Xun et al. (2012) and used to assess the level of workplace bullying in nurses. The questionnaire contained 22 items that measured three dimensions including work‐related bullying, person‐related bullying, and organization‐related bullying. Each item was scored ranging from 1 (never) to 5 (every day), with higher scores indicating more serious workplace bullying. The scale content validity index CVI was 0.919, the internal consistency coefficient was 0.915, and the test–retest reliability was 0.883 (Xun et al., 2012).

#### 3.3.3 Workplace spirituality

The Workplace Spirituality Scale (WSS) developed by Ke et al. (2014) was used to assess the level of workplace spirituality in nurses. The questionnaire contained 27 items that measured three dimensions including meaningfulness of work, team spirit, and organizational values. Each item was scored ranging from 1 (strongly disagree) to 4 (strongly agree). A higher score indicates higher levels of workplace spirituality. The internal consistency coefficient of scale was 0.90, and the test–retest reliability was 0.77 (Ke et al., 2014).

#### 3.3.4 Job burnout

The Chinese version of the Maslach Burnout Inventory‐General Survey (MBI‐GS), originally developed by Maslach et al. (2001), was translated into Mandarin Chinese by Li et al. (2003) and used to assess the level of job burnout in nurses. The questionnaire contained 15 items that measured three dimensions including emotional exhaustion, depersonalization, or dehumanization, and low levels of personal accomplishment. Each item was scored ranging from 0 (never) to 6 (very frequently). A higher score indicates higher levels of job burnout. This tool has previously demonstrated acceptable internal consistency (Cronbach's *a* = 0.732) and predictive validity in a sample of nurses (Zhu et al., 2007).

### 3.4 Ethical considerations

All data in this research were anonymous, stored in a password‐protected computer and only available to members of the research team. Researchers were forbidden from disclosing the personal information of participants to ensure their privacy. The study was approved by the Ethics Committee of Xiangyang No.1 People's Hospital, Hubei University of Medicine (NO. 2020KY033‐05).

### 3.5 Data analysis

We conducted descriptive statistics, correlations, and multiple regression by using IBM SPSS v23.0. Chi‐square and *t* tests were used to compare the differences in paediatric nurses using the scores for workplace bullying, workplace spirituality, and job burnout. The data were normally distributed in the study; thus, Pearson's correlation coefficient (*r*) was used to conduct correlation analysis for the study variables. Multiple linear regression analysis was used to explore the independent factors of job burnout.

The testing of the hypothesized model was conducted using AMOS version 23.0. SEM with maximum likelihood estimation was used to test the fit between the data and the hypothesized model. The following indices were used to test the hypothesized mediational models (McDonald & Ho, 2002): Chi‐square (*χ* ^2^) and the Chi‐square/degrees of freedom ratio (*χ* ^2^/df), Root Mean Square Error of Approximation (RMSEA) ≤ 0.08, Comparative Fit Index (CFI) ≥ 0.09, and Incremental Fit Index (IFI) ≥ 0.09. We followed Preacher and Hayes' (Preacher & Hayes, 2008) approach to test the mediation model and used 5000 sample sizes recommended by Preacher and Hayes (Preacher & Hayes, 2008) in the bootstrap method.

## 4 RESULTS

### 4.1 Characteristics of workplace bullying, workplace spirituality, and job burnout in paediatric nurses

A total of 485 paediatric nurses were distributed online questionnaires for this survey, and 465 paediatric nurses responded and completed the questionnaire. The 63 invalid questionnaires were excluded due to obviously insufficient time to complete the survey. Finally, the data of 402 paediatric nurses were analysed. The majority of nurses were female (97.76%), averaging 33 years of age with 11.14 years nursing experience. The distributions of workplace bullying, workplace spirituality, and job burnout in the paediatric nurses are shown in Table 1.

**TABLE 1:** Demographic information of the study participants and the distributions of workplace bullying, workplace spirituality, job burnout by observed variables.

Variable	*n* (%)	WB mean ± SD	WS mean ± SD	JB mean ± SD
Gender				
Male	9 (2.24)	1.85 ± 0.70	2.12 ± 0.52	2.80 ± 1.32
Female	393 (97.76)	1.72 ± 0.47	2.87 ± 0.48	2.79 ± 1.14
*p*		0.586	<0.001***	0.985
*t*		0.567	−4.601	0.019
Age				
≤30	180 (44.78)	1.67 ± 0.43	2.80 ± 0.46	2.75 ± 1.67
31 ~ 40	154 (38.31)	1.70 ± 0.42	2.84 ± 0.53	2.97 ± 1.17
≥41	68 (16.91)	1.92 ± 0.62	2.99 ± 0.50	2.50 ± 0.94
*p*		0.001**	0.027*	0.015*
*F*		7.556	3.653	4.268
Marital status				
Single	109 (27.11)	1.70 ± 0.46	2.79 ± 0.47	2.95 ± 1.18
Married	293 (72.89)	1.73 ± 0.49	2.87 ± 0.50	2.73 ± 1.12
*p*		0.562	0.122	0.083
*t*		−0.580	−1.550	1.735
Years of experience				
≤5	126 (31.34)	1.64 ± 0.44	2.80 ± 0.43	2.71 ± 1.19
6 ~ 10	103 (25.62)	1.70 ± 0.39	2.82 ± 0.50	2.87 ± 1.09
11 ~ 15	99 (24.63)	1.76 ± 0.48	2.82 ± 0.54	3.06 ± 1.23
≥16	74 (18.41)	1.86 ± 0.60	3.01 ± 0.50	2.46 ± 0.91
*p*		0.012*	0.017*	0.005**
*F*		3.676	3.438	4.384
Employment type				
Permanent nurse	78 (19.40)	1.79 ± 0.53	3.00 ± 4.81	2.51 ± 1.00
Contract nurse	324 (80.60)	1.71 ± 0.46	2.81 ± 0.49	2.86 ± 1.17
*p*		0.158	0.002**	0.008**
*t*		1.415	9.382	−2.705
Education				
Technical secondary school	6 (1.49)	2.03 ± 0.86	2.72 ± 0.61	1.90 ± 0.84
Junior college	47 (11.69)	1.72 ± 0.54	2.87 ± 0.41	2.61 ± 1.05
Undergraduate	329 (81.84)	1.71 ± 0.44	2.86 ± 0.50	2.81 ± 1.14
Postgraduate	20 (4.98)	1.91 ± 0.65	2.66 ± 0.58	3.12 ± 1.32
*p*		0.115	0.329	0.090
*F*		1.986	1.150	2.179
Title				
Nurses	85 (21.15)	1.67 ± 0.44	2.80 ± 0.45	2.69 ± 1.13
Senior nurses	174 (43.28)	1.69 ± 0.39	2.83 ± 0.45	2.90 ± 1.21
Supervisor nurses	117 (29.10)	1.79 ± 0.56	2.83 ± 0.57	2.77 ± 1.12
Deputy chief nurse or above	26 (6.47)	1.87 ± 0.61	3.21 ± 0.47	2.51 ± 0.75
*p*		0.090	0.001**	0.289
*F*		2.181	5.280	1.256
Shift type				
Day shift	162 (40.30)	1.69 ± 0.52	2.92 ± 0.48	2.63 ± 1.05
Three shift	240 (59.70)	1.75 ± 0.44	2.80 ± 0.50	2.90 ± 1.19
*p*		0.187	0.020*	0.016*
*t*		−1.320	2.331	−2.430

*Note*: *N* = 402; *p < 0.05, **p < 0.01, ***p < 0.001.

Abbreviations: JB, Job burnout; SD, Standard Deviation; WB, Workplace bullying; WS, Workplace spirituality.

### 4.2 Correlation analysis

According to correlation analysis, we found that the workplace bullying of paediatric nurses was significantly and positively correlated to job burnout (*r* = 0.459, *p* < 0.001). Bullying was significantly and negatively correlated to workplace spirituality (*r* = −0.187, *p* < 0.001). Workplace spirituality was significantly and negatively correlated to job burnout (*r* = −0.206, *p* < 0.001).

### 4.3 Multiple linear regression analysis of the factors of job burnout among the observed paediatric nurses

To explore factors affecting job burnout in paediatric nurses, a multiple linear regression model of the impact factors associated with job burnout was constructed. Multiple linear regression analysis was performed taking the job burnout score as a dependent variable and the statistically significant variables in the univariate analysis, workplace spirituality score and workplace bullying as independent variables (Table 2). The results showed that workplace bullying, and workplace spirituality were the influencing factors of job burnout in paediatric nurses.

**TABLE 2:** Results of multiple stepwise linear regression analysis of the factors of job burnout in the paediatric nurses.

	B	SE	Beta	*t*	95% CI	*p*
Age	0.016	0.025	0.104	0.648	(−0.033, 0.066)	0.517
Years of experience	−0.022	0.022	−0.163	−1.002	(−0.065, 0.021)	0.317
Employment type: Contract nurse	0.288	0.158	0.100	1.824	(−0.022, 0.599)	0.069
Shift type: Three shift	0.031	0.119	0.013	0.260	(−0.203, 0.264)	0.795
Workplace bullying	1.107	0.113	0.459	9.795	(0.885, 1.329)	<0.001***
Workplace spirituality	−0.221	0.105	−0.096	−2.108	(−0.427, −0.015)	0.036*

*Note*: *N* = 402; *p < 0.05, **p < 0.01, ***p < 0.001.

Abbreviation: SE, standard error.

### 4.4 Structural equation modelling results

**FIGURE 2:**
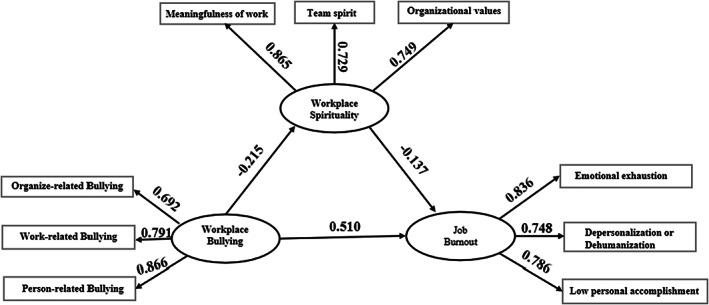
Path coefficient of structural equation model (Standardized coefficients).

The initial SEM model exhibited a reasonable fit to the data (*χ* ^*2*^ = 53.699, df = 24, *p* < 0.001, *χ* ^2^/df = 2.237, CFI = 0.980, IFI = 0.980, RMSEA = 0.056). The estimates for structural paths are shown in Table 3, and the path coefficients for the structural equation model are shown in Figure 2. We also tested the mediating role of workplace spirituality in the relationship between workplace bullying and job burnout. The results are shown in Table 4; both 95% CI (0.008, 0.093) in indirect effect and 95% CI (0.502, 0.863) in direct effect did not cross 0, which indicated that workplace spirituality partially mediated the relationship between workplace bullying and job burnout. The ratio of mediating effect with total effect was 5.38%.

**TABLE 3:** Effect estimates for hypothesized structural paths.

Structural paths	Unstandardized coefficients	Standardized coefficients	SE	Critical ratio	*p*
WB → WS	−0.173	−0.215	0.048	−3.616	<0.001
WS → JB	−0.323	−0.137	0.129	−2.510	0.012
WB → JB	0.963	0.510	0.119	8.122	<0.001

Abbreviations: SE, standard error; WB, Workplace bullying; WS, Workplace spirituality.

**TABLE 4:** Mediating model and the mediation effect of workplace spirituality.

	Effect	SE	*p*	95% CI
Indirect effect	0.029	0.021	0.009	(0.008, 0.093)
Direct effect	0.510	0.093	<0.001	(0.502, 0.863)
Total effect	0.539	0.093	<0.001	(0.542, 0.908)

Abbreviation: SE, standard error.

## 5 DISCUSSION

The results of this study support the proposition that workplace spirituality is associated with paediatric nurses experiencing bullying and burnout. Workplace spirituality may reduce workplace bullying and burnout by changing perceptions and evaluations of workplace bullying in paediatric nurses.

Our study showed that the score of job burnout among paediatric nurses was 2.79 ± 1.14, which was similar to the results of previous studies (Kim et al., 2019; Zhou et al., 2017). The results of multiple linear regression analysis showed that workplace bullying and workplace spirituality were influencing factors of burnout. Consistent with previous studies on workplace bullying, bullying is associated with high levels of burnout (Kim et al., 2019; Spence Laschinger et al., 2012; Yun & Kang, 2018). Continuous exposure to stressors in the workplace is an important mechanism for the development of severe burnout (Maslach & Leiter, 1997). Bullying in the workplace is a progressive, cumulative, and highly personalized harmful experience, and it is an important factor in the occurrence of job burnout among nurses (Etienne, 2014). Kinjerski (2013) proposed that workplace spirituality is a positive state that includes physical, affective, cognitive, interpersonal, spiritual, and mystical dimensions. There is evidence (Pirkola et al., 2016) that workplace spirituality can enhance job involvement and organizational commitment, which are important in improving an organization's effectiveness and productivity and in reducing workplace deviance and turnover rate (Amin et al., 2020).

In this study, workplace bullying of paediatric nurses was at a low‐moderate level, and organization‐related bullying scored the highest, which is consistent with previous research results (Chen et al., 2021; Lin et al., 2017). Nurses generally believed that they have undertaken an overloaded workload and their due rights were not guaranteed, such as statutory rest and sick leave (Chen & Zheng, 2020). Our research showed that nurses who had worked more years and were higher in seniority were more likely to suffer from workplace bullying, which is inconsistent with previous research results (Spence Laschinger et al., 2012). To explain the results of our study, it is necessary to understand the workplace situation for senior nurses. First, senior nurses have a wealth of experience. In addition to being given routine clinical work, they also undertake multiple tasks such as teaching and management. They have been in a high‐intensity and overloaded work environment for a long time, which is likely to cause greater psychological and work pressures (Luan et al., 2017). Second, some managers may think that senior nurses are not as easy to manage as lower senior nurses, and they do not see the advantages of senior nurses. The managers may have a discriminatory attitude towards senior nurses, depriving them of their main job responsibilities, and arranging for them to perform trivial tasks that are below their level of ability (Wang et al., 2016; Zhang & Shang, 2015). Therefore, hospital managers should acknowledge that workplace bullying exists, and make efforts to rationally allocate human resources, reduce nurses' work pressure, and ensure that the rights and interests of nurses are met.

The nursing profession is essentially service‐oriented. Nurses often face many challenges (Arnetz et al., 2020), such as death of patients, public health emergencies, and work overload. Hospitals often represent an unbalanced working environment with little room for change (Hayashi et al., 2020). Even with such a work environment, the workplace spirituality of paediatric nurses in this study was surprisingly high, which may be related to the nobility of their profession. Past studies have shown that there is a positive correlation between a sense of belonging and workplace spirituality (van der Walt & de Klerk, 2014). In our study, for paediatric nurses who were permanently employed by their hospitals, it was observed that the older the age and the longer the working hours, the higher their level of workplace spirituality. One possible explanation is that this situation creates a higher sense of belonging where the employees work together with the hospital to achieve a common goal (Zhang et al., 2017). Another possible explanation is that only by identifying with one's own profession, can they be dedicated to it for a long time (Niskala et al., 2020). In this study, the workplace spirituality of postgraduate nurses was significantly lower than that of associates and undergraduates, which is inconsistent with previous research results (Ke et al., 2017). Analysis of the reason may be that nursing graduate students have a wider scope and choice of employment (Wang et al., 2010), which reduces their sense of belonging. On the other hand, it may be related to the fact that nursing graduate students have not yet adjusted their understanding of clinical nursing and the realities of the job, leading to excessive professional expectations and a reduction in the obtained professional value (Li et al., 2020).

In Figure 2, the SEM shows that workplace bullying had a negative influence on paediatric nurses workplace spirituality and positive influence on job burnout. Workplace bullying influenced burnout indirectly through workplace spirituality, highlighting the fundamental importance of workplace bullying in paediatric nurses burnout. The mediating effect in our study reveals that workplace spirituality partially mediates the relationship between workplace bullying and job burnout. Previous studies have shown (Spence Laschinger et al., 2012) that a healthy working environment is an important factor influencing nurse retention. Workplace spirituality, as a positive inner experience, is conducive to creating a good working environment and atmosphere, encouraging interpersonal communication between employees, and increasing employees' ability to understand others, thereby reducing the occurrence of uncivilized behaviour in the workplace (Phillips et al., 2018). When faced with workplace bullying, workplace spirituality can help nurses to correctly recognize and evaluate stress, thereby generating positive benefits for individuals, teams, and organizations, effectively improving nurses' job involvement, physical health, job satisfaction and retention intention, and reducing work stress in the environment (Kazemipour et al., 2012; Schneider et al., 2015).

The results of our study reveal the internal mechanisms and impact that workplace spirituality in paediatric nurses has on bullying and job burnout. The findings support existing theories about workplace spirituality, bullying, and job burnout, and provide new information on how these concepts are interrelated to explain the job burnout of paediatric nurses.

## 6 LIMITATIONS

This study is the first to report evidence suggesting that workplace spirituality may reduce the occurrence of workplace bullying in paediatric nurses. Our study also has some limitations. First, for the sake of convenience, we only sampled paediatric nurses from just one province, so the sample representation was not adequate. Second, data collection relied on self‐reporting by the paediatric nurses. Therefore, we cannot guarantee that the self‐reported results of each participant were accurate. Finally, our model shows the factors related to job burnout in paediatric nurses. However, we admit that there are many other factors that can explain the job burnout of paediatric nurses, which were not measured in this study. In addition to workplace spirituality, other factors such as personality characteristics and social support may also play an important role in the job burnout of paediatric nurses. These limitations should be addressed in further research.

## 7 CONCLUSIONS

Our study has contributed to the body of knowledge pertaining to mechanisms of job burnout. The results of this study highlight the importance of workplace spirituality for self‐stress evaluation and cognition, which may reduce the incidence of workplace bullying, thereby reducing the job burnout of paediatric nurses.

## 8 RELEVANCE TO CLINICAL PRACTICE

This study provides new perspectives and dimensions for nursing management, which requires urgent attention from nursing managers and researchers. Nursing managers should assist in cultivating and developing the workplace spirit of paediatric nurses as part of a program to eliminate workplace bullying and reduce job burnout, so as to maintain the stability of the nursing team in the face of an extreme shortage of paediatric nurses.

## AUTHOR CONTRIBUTIONS

Study conception and design: Ying Fan and Limin Xing; Data collection: Ying Fan, Mi Cao, Yumei Zhou and Peng Duan; Data analysis and interpretation: Ying Fan and Yumei Zhou; Drafting of the article: Ying Fan and Limin Xing; Critical revision of the article: Limin Xing. All the authors have carefully reviewed the article and approved the final draft.

## FUNDING INFORMATION

This study was not funded.

## CONFLICT OF INTEREST STATEMENT

The authors have declared that there is no conflict of interest.

## Data Availability

The datasets generated during and/or analysed during the current study are not publicly available due to confidentiality reason but are available from the corresponding author on reasonable request.
